# Predictive Factors of Death and the Clinical Profile of Hospitalized Covid-19 Patients in Morocco: A One-Year Mixed Cohort Study

**DOI:** 10.7759/cureus.32462

**Published:** 2022-12-13

**Authors:** Faïza Charif, Zaynab Mahdi, Fadila Bousgheiri, Hassana Belafki, Adil Gourinda, Karima Sammoud, Fadila Salmane, Wiam Ftouh, Mariem Benkacem, Adil Najdi

**Affiliations:** 1 Department of Epidemiology and Public Health, Mohamed VI University Hospital of Tangier, Tangier, MAR; 2 Department of Endocrinology, Diabetes, and Metabolic Diseases, Mohamed VI University Hospital of Tangier, Tangier, MAR

**Keywords:** predictors, clinical profile, sars-cov-2, mortality, covid-19

## Abstract

Background

Since the onset of the Covid-19 pandemic, several studies have been conducted around the world in an attempt to understand this heterogeneous and unpredictable disease and to prevent related death. It was therefore necessary to study the associated risk factors of Covid-19-related mortality.

Objectives

The aim of this study was to describe the clinical profile and to identify the factors associated with mortality of patients with Covid-19 in Morocco.

Methods

We performed a mixed cohort study (retrospective and prospective) of 615 in-patients with Covid-19 disease, enrolled between August 2020 and October 2021. We followed the cohort throughout the hospitalization until discharge and 30 days thereafter.

Results

The median age was 64 years old; 62.1% of the patients were male. The mean time from symptom onset to hospitalization was 8.5 days (±4.67), and 68.1% of patients had comorbidities. On admission, the most common symptoms were dyspnea (82.2%), cough (80.3%), and fever (76.8%). The main follow-up complication was secondary infection (56.9%). Based on univariate analysis, male gender (p<0.008 and brut relative risk {_b_RR}=1.57), advanced age (p<0.001), lung involvement (p<0.001), lymphopenia (p<0.001 and _b_RR=2.32), D-dimers of >500 µg/l (p<0.007 and _b_RR=2.47), C-reactive protein (CRP) of >130 mg/l (p<0.001 and _b_RR=2.45), elevated creatinine (p<0.013 and _b_RR=1.61), lactate dehydrogenase (LDH) of >500 U/l (p<0.001 and _b_RR=7.16), receiving corticosteroids (p<0.001 and _b_RR=5.08), invasive ventilation (p<0.001 and _b_RR=30.10), the stay in the resuscitation unit (p<0.001 and _b_RR=13.37), and acute respiratory distress syndrome (ARDS) (p<0.001 and _b_RR=10.98) were associated with a higher risk of death. In the opposite, receiving azithromycin and hydroxychloroquine (p<0.001 and _b_RR=0.28) and pre-admission anticoagulants (p<0.005 and _b_RR=0.46) was associated with a lower risk of mortality. Multivariate regression analysis showed that age of >60 years (p<0.001 and adjusted odds ratio {aOR}=4.90), the use of invasive ventilation (p<0.001 and aOR=9.60), the stay in the resuscitation unit (p<0.001 and aOR=5.09), and acute respiratory distress syndrome (p<0.001 and aOR=6.49) were independent predictors of Covid-19 mortality.

Conclusion

In this cohort study focusing on Covid-19 in-patient's mortality, we found that age of >60 years, the use of invasive ventilation, the stay in the resuscitation unit, and acute respiratory distress syndrome were independent predictors of Covid-19 mortality. The results of this study can be used to improve knowledge for better clinical management of Covid-19 in-patients.

## Introduction

Since the beginning of 2020, the world has experienced the spread of a new virus called SARS-CoV-2, causing a pandemic. This outbreak has had enormous health repercussions in terms of morbidity and mortality, as it has affected (until March 23, 2022) more than 472 million people worldwide, including more than six million deaths [1]. In Morocco, the first case of the disease was detected on March 2, 2020, and a state of emergency was declared in the country on March 23, 2020. Despite the relentless efforts of the authorities to stop the spread of the virus, the country has not avoided the epidemic, and by the end of March 2022, more than 1.1 million subjects were infected, with more than 1,650 deaths [2]. International clinical observations since the beginning of the pandemic have shown that the disease is heterogeneous and unpredictable. It has taken clinical forms ranging from asymptomatic to very severe and fatal. In an effort to understand the disease and to prevent mortality, research has been initiated worldwide. Many determinants of mortality have been identified in studies, such as advanced age [3], male sex, and the presence of comorbidities [4]. It was therefore necessary to study the mortality-related factors in Morocco, in order to have a better understanding of Covid-19 in-patient's management. The aim of this study was to describe the clinical profile and to identify the predictive factors of mortality of in-patients with Covid-19 in Morocco.

## Materials and methods

Study design, participants, and eligibility criteria

We performed a mixed cohort study (retrospective and prospective) of in-patients with Covid-19 disease. The target population was SARS-CoV-2-infected in-patients in Morocco. Our sample included in-patients diagnosed between August 2020 and September 2021, in the Tangier-Tetouan-Al Hoceima region.

Inclusion Criteria

The inclusion criteria were as follows: patients over 18 years old; SARS-CoV-2 infection confirmed by either reverse transcription-polymerase chain reaction (RT-PCR), rapid antigenic test, or CT scan; and in-patients admitted in Covid-19 care units according to the Ministry of Health criteria (asymptomatic or mild Covid-19 clinical form with one or more risk factors; moderate, severe, or critical cases; and mild cases initially managed on an outpatient basis and whose condition has worsened) [5].

Noninclusion Criteria

The noninclusion criteria were as follows: SARS-CoV-2-positive patients with asymptomatic or mild form, non-consenting patients, and those for whom follow-up after discharge was impossible.

Enrollment Period

Inclusion in the study was performed using a mixed strategy in parallel. A retrospective inclusion was performed using the medical records of patients meeting the inclusion criteria admitted between August 2020 and January 2021. And simultaneously, a prospective enrollment included patients newly admitted to the hospital between February 2021 and October 2021.

Sample size

For a minimum needed number of deaths set at 60, we calculated a sample size of 400 in-patients (considering an in-patient mortality rate of 15%) [6].

Data collection

The International Severe Acute Respiratory and Emerging Infection Consortium (ISARIC)-WHO Case Report Form (CRF) in its core form [7] was modified and adapted to our context to develop the CRF for our study. This CRF consists of four modules: Module 1 was completed on the first day of hospitalization; Module 2 was completed at day 7 (D7), D14, D21, and D28 of hospitalization; Module 3 was completed at discharge or after death; and Module 4 was completed for patients discharged alive, at D15 and D30 after discharge.

In each module, we have collected the following data: Module 1: sociodemographic data, vaccination, date of onset and vital parameters at admission, comorbidities and risk factors, pre-admission medications, signs and symptoms at admission, paraclinical data at admission (results of PCR and/or rapid antigen test, biological tests, CT scan, and ECG), supportive care, and treatment started; Module 2: vital parameters, daily clinical characteristics, biological test results, supportive care, treatment received, complications, and patient status at the end of the week; Module 3: outcome information and date of outcome; and Module 4: evolution after discharge (vital status).

Follow-up

Patients were followed throughout their hospitalization until discharge. We followed the progress of patients discharged alive by phone call at D15 and D30 after discharge to know the vital status.

Statistical analysis

First, we performed a descriptive analysis. For quantitative variables, we calculated means, and for categorical variables, we calculated frequencies and 95% confidence intervals (95% CI). Next, a univariate analysis compared categorical variables with the chi-square test (p<0.05) and relative risk with 95% confidence intervals. To adjust on confounding factors, a multivariate analysis was performed including all variables with p<0.20 and using a binary logistic regression model. All statistical analyses were performed with Statistical Package for Social Sciences (SPSS) (IBM SPSS Statistics, Armonk, NY) version 21 software.

Ethical issues

Informed consent was obtained from each in-patient included in the study. Data were collected anonymously. The study received ethical approval from "Fes University Hospital Ethics Committee" (approval number: 03/21), a Moroccan ethics committee.

## Results

Sociodemographic description

We analyzed 615 confirmed hospitalized cases with Covid-19 in total. The mean age was 62.53±13.67 years old, and the median age was 64 years old. Baseline information including sociodemographic and tobacco status is presented in Table 1.

**Table 1:** Sociodemographic characteristics of Covid-19 patients

General characteristics	Number (%)
Sex	
Male	382 (62.1)
Female	233 (37.9)
Age group	
18-39	38 (6.2)
40-49	69 (11.2)
50-59	110 (17.9)
60-69	207 (33.7)
70-79	129 (21.0)
80 and more	62 (10.1)
Residence area	
Urban	603 (98.0)
Rural	12 (2.0)
Smoking	
Active smoker	31 (5.7)
Nonsmoker	517 (94.3)

Clinical outcome

The patients in our sample were admitted to three different units according to the severity of their condition on admission: 76 (12.4%) were admitted to the intermediate care unit, 343 (55.8%) to the intensive care unit (ICU), and 196 (31.9%) to the resuscitation unit. Regarding their vaccination status, 80 (13.4%) patients (n=598) were vaccinated, of whom 13 had received one dose and 67 had received two doses. Of the 233 female patients, six were pregnant.

The mean time from symptom onset to hospitalization was 8.5 days (±4.67), and the median time was eight days. On admission, the mean of oxygen saturation on room air was 78.61%±14.98%, and the mean of saturation after oxygen therapy was 90.74%±8.99%. Table 2 presents the medical history of our cohort, the symptoms, and the laboratory parameters at admission.

**Table 2:** Clinical characteristics among in-patients with Covid-19 (n=615) ^a^Creatinine of >13 mg/l for males and >10 mg/l for females CRP: C-reactive protein; LDH: lactate dehydrogenase

Variables	Yes (number {%})	No (number {%})
Comorbidities		
Anemia	224 (39.6)	342 (60.4)
Hypertension	217 (35.7)	390 (64.3)
Chronic respiratory disease	11 (1.8)	597 (98.2)
Asthma	21 (3.4)	588 (96.6)
Diabetes	308 (50.7)	299 (49.3)
Chronic kidney failure	35 (5.8)	572 (94.2)
Chronic hepatic failure	1 (0.2)	608 (99.8)
Chronic neurological disorder	10 (1.6)	598 (98.4)
Malign neoplasm	11 (1.8)	597 (98.2)
Dysthyroid disease	28 (4.6)	581 (95.4)
Obesity	105 (33.9)	205 (66.1)
Onset symptoms		
Fever	466 (76.8)	141 (23.2)
Cough	484 (80.3)	119 (19.7)
Dyspnea	498 (82.2)	108 (17.8)
Sore throats	46 (7.7)	549 (92.3)
Wheezing	10 (1.7)	576 (98.3)
Thoracic pain	213 (35.3)	390 (64.7)
Subcostal printing	53 (8.9)	545 (91.1)
Anosmia	90 (15.2)	501 (84.8)
Ageusia	49 (8.3)	541 (91.7)
Conjunctivitis	2 (0.3)	599 (99.7)
Lymphadenopathy	4 (0.7)	591 (99.3)
Headaches	295 (49.0)	307 (51.0)
Convulsions	9 (1.5)	596 (98.5)
Fatigue/discomfort	279 (46.1)	326 (53.9)
Confusion	44 (7.3)	561 (92.7)
Myalgias	80 (13.3)	520 (86.7)
Arthralgias	57 (9.5)	540 (90.5)
Diarrhea	132 (22.0)	469 (78.0)
Emesis/nauseas	147 (24.4)	455 (75.6)
Bleeding	4 (0.7)	599 (99.3)
Laboratory parameters at admission		
Lymphocytes of <1500	340 (78.5)	93 (21.5)
High blood creatinine^a^	166 (33.5)	329 (66.5)
D-dimers of >500 µg/l	254 (82.7)	53 (17.3)
CRP of >130 mg/l	193 (47.7)	212 (52.3)
Ferritin of >300 µg/l	136 (77.3)	40 (22.7)
LDH of >500 U/l	83 (49.7)	84 (50.3)

Regarding pre-admission medication, 8.6% (52) of patients were on angiotensin-converting enzyme (ACE) inhibitors, 12.5% (75) on angiotensin receptor blockers (ARBs), 6.1% (37) on statins, 9.7% (59) on antiplatelets, and 11.5% (70) on anticoagulants. Regarding the patient's lung involvement, admission CT scan showed minimal to moderate involvement (<25%) in 8.6%, extensive involvement (25%-50%) in 30.4%, severe involvement (50%-75%) in 37.0%, and critical involvement (>75%) in 24.1%.

Regarding treatment, 32.2% of patients received azithromycin alone, 2.5% received hydroxychloroquine alone, 55.8% received the combination of azithromycin and hydroxychloroquine, and 9.4% received neither hydroxychloroquine nor azithromycin. Of the patients, 94% received vitamin C, 74.0% received vitamin D, and 90.7% received zinc. Moreover, 95.2% (570) of patients received low-molecular-weight heparin (LMWH), 72.8% (259) received corticosteroids, and 56.9% received additional antibiotic therapy (besides azithromycin). During hospitalization, 18.9% of patients (116) received invasive ventilation, and 37.2% (229) has been through the resuscitation unit.

Overall description of the evolution

During the hospitalization period, patients experienced complications, the main ones of which are presented in Table 3. The average length of hospital stay was 6.61 days (±5.65).

**Table 3:** Complications during hospitalization

Complications	Yes (number {%})	No (number {%})
Acute respiratory distress syndrome	94 (26.1)	266 (73.9)
Pulmonary embolism	70 (19.8)	283 (80.2)
Secondary infection	350 (56.9)	265 (43.1)
Acute kidney failure	25 (8.6)	265 (91.4)
Hepatic cytolysis	85 (19.5)	351 (80.5)

**Figure 1:**
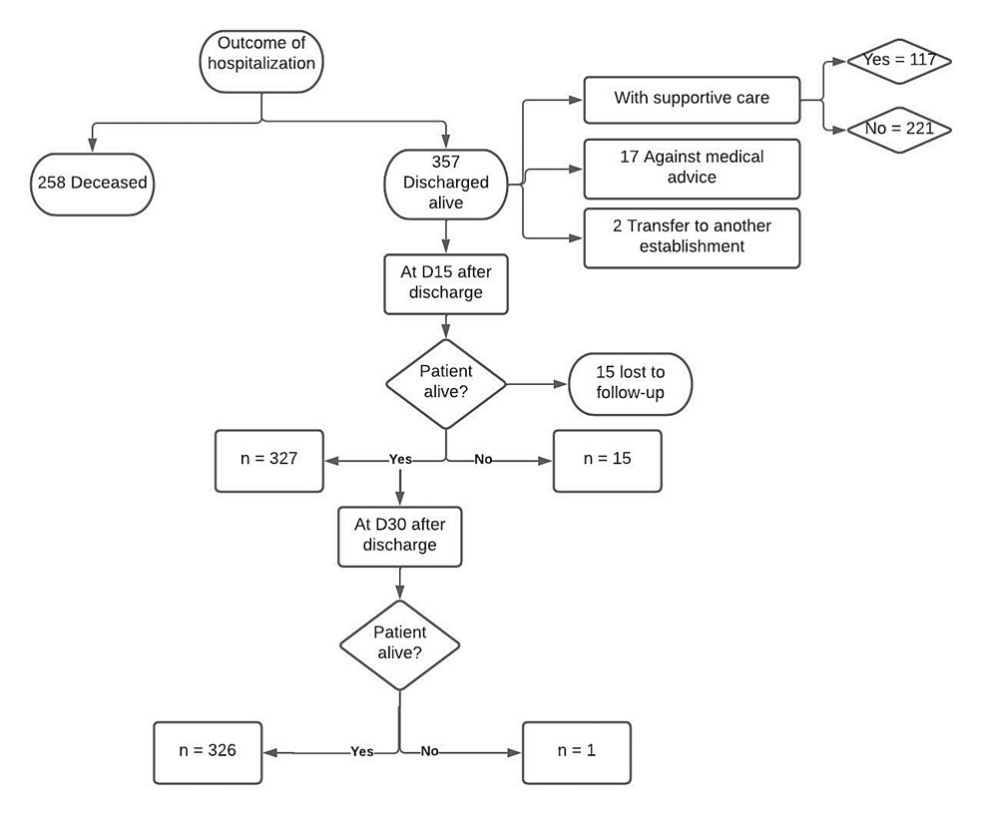
Flowchart of patients from enrollment to the end of follow-up: a mixed cohort study of 615 Covid-19 in-patients from Tangier, Morocco D15: day 15; D30: day 30

The endpoint during the follow-up period was death by Covid-19. During hospitalization, 258 patients died, and 357 were discharged alive. Within 30 days of discharge, we noted 16 additional deaths. In total, at the end of follow-up, of the 615 patients hospitalized with Covid-19, 44.6% (95% CI: 40.6-48.6) died from the disease, 53% (95% CI: 49.0-57.0) survived, and 2.4% were lost to follow-up. Figure 1 details patient outcomes at discharge and their progress 30 days after leaving the hospital.

Predictors of Covid-19 mortality: univariate analysis

The results of the univariate analysis are reported in Table 4.

**Table 4:** Mortality according to sociodemographic and clinical characteristics among in-patients with Covid-19 (n=615) _b_RR: brut relative risk; ACE: angiotensin-converting enzyme; ARBs: angiotensin receptor blockers; CRP: C-reactive protein; LDH: lactate dehydrogenase; LMWH: low-molecular-weight heparin 95% CI: 95% confidence interval

	Deceased patients			
Variable	Number (%)	95% CI	P-value	_b_RR
Sex (n=600)			<0.008	
Male	187 (49.9)	44.9-54.9		1.57
Female	87 (38.7)	32.3-45.1		1
Age range (n=600)			<0.001	
18-39	6 (15.8)	6.0-31.3		
40-49	22 (32.4)	21.5-44.8		
50-59	37 (34.3)	25.4-44.0		
60-69	97 (48.7)	41.6-55.9		
70-79	67 (52.8)	43.7-61.7		
80 and more	45 (75.0)	62.1-85.3		
Vaccination (n=583)			0.805	
Yes	37 (47.4)	36.0-59.1		1.06
No	232 (45.9)	41.5-50.4		1
Anemia (n=552)			0.550	
Yes	103 (47.0)	40.3-53.9		1.11
No	148 (44.4)	39.0-50.0		1
Chronic cardiopathy (n=590)			0.052	
Yes	38 (56.7)	44.0-68.8		1.65
No	231 (44.2)	39.9-48.5		1
Hypertension (n=592)			0.625	
Yes	98 (46.4)	39.6-53.4		1.08
No	169 (44.4)	39.3-49.5		1
Asthma (n=594)			0.263	
Yes	7 (33.3)	14.6-57.0		0.59
No	262 (45.7)	41.6-49.9		1
Chronic kidney failure (n=592)			0.703	
Yes	16 (48.5)	30.8-66.5		1.14
No	252 (45.1)	40.9-49.3		1
Dysthyroid disease (n=594)			0.502	
Yes	11 (39.3)	21.5-59.4		0.76
No	259 (45.8)	41.6-50.0		1
Diabetes (n=592)			0.395	
Yes	140 (46.8)	41.1-52.7		1.15
No	127 (43.3)	37.6-49.2		1
Obesity (n=306)			0.550	
Yes	33 (31.7)	22.9-41.6		0.85
No	71 (35.1)	28.6-42.2		
Smoking (n=537)			0.635	
Yes	15 (50.0)	31.3-68.7		1.19
No	231 (45.6)	41.2-50.0		1
Pre-admission ACE inhibitors (n=587)			0.774	
Yes	24 (47.1)	32.9-61.5		1.08
No	241 (45.0)	40.7-49.3		1
Pre-admission ARBs (n=587)			0.376	
Yes	29 (40.3)	28.9-52.5		0.79
No	236 (45.8)	41.5-50.2		1
Pre-admission statins (n=593)			0.345	
Yes	19 (52.8)	35.5-69.6		1.38
No	249 (44.7)	40.5-48.9		1
Pre-admission antiplatelets (n=594)			0.226	
Yes	22 (37.9)	25.5-51.6		0.71
No	248 (46.3)	42.0-50.6		1
Pre-admission anticoagulants (n=594)			<0.005	
Yes	20 (29.4)	19.0-41.7		0.46
No	250 (47.5)	43.2-51.9		1
Lung involvement (n=591)			<0.001	
Minimal to moderate, <25%	8 (15.4)	6.9-28.1		
Extensive, 25%-50%	62 (34.6)	27.7-42.1		
Severe, 50%-75%	95 (43.6)	36.9-50.4		
Critical, >75%	103 (72.5)	64.4-79.7		
Lymphocytes of <1500 (n=421)			<0.001	
Yes	182 (55.3)	49.8-60.8		2.32
No	32 (34.8)	25.1-45.4		1
D-dimers of >500 µg/l (n=300)			<0.007	
Yes	112 (45.2)	38.9-51.6		2.47
No	13 (25.0)	14.0-38.9		1
CRP of >130 mg/l (n=393)			<0.001	
Yes	113 (59.8)	52.4-66.8		2.45
No	77 (37.7)	31.1-44.8		1
High blood creatinine (n=482)			0.013	
Yes	90 (55.2)	47.2-63.0		1.61
No	138 (43.3)	37.8-48.9		1
Ferritin of >300 µg/l (n=169)			0.31	
Yes	73 (56.6)	47.6-65.3		1.44
No	19 (47.5)	31.5-63.9		1
LDH of >500 U/l (n=160)			<0.001	
Yes	61 (77.2)	66.4-85.9		7.16
No	26 (32.1)	22.2-43.4		1
Received protocol (n=578)			<0.001	
Azithromycin alone	87 (46.8)	39.4-54.2		
Hydroxychloroquine alone	3 (20.0)	4.3-48.1		
Combination azithromycin+hydroxychloroquine	135 (41.8)	36.4-47.4		
None	39 (72.2)	58.4-83.5		
LMWH (n=584)			0.352	
Yes	248 (44.6)	40.4-48.8		0.69
No	15 (53.6)	33.9-72.5		1
Corticosteroids (n=346)			<0.001	
Yes	104 (41.8)	35.6-48.2		5.08
No	12 (12.4)	6.6-20.6		1
Invasive ventilation (n=600)			<0.001	
Yes	109 (94.0)	88.0-97.5		30.10
No	165 ( 34.1)	29.9-38.5		1
Stay in the resuscitation unit (n=600)			<0.001	
Yes	182 (81.3)	75.5-86.1		13.37
No	92 (24.5)	20.2-29.1		1
Pulmonary embolism (n=344)			0.193	
Yes	27 (39.1)	27.6-51.6		1.43
No	85 (30.9)	25.5-36.7		1
Acute respiratory distress syndrome (n=350)			<0.001	
Yes	68 (73.1)	62.9-81.8		10.98
No	51 (19.8)	15.1-25.3		1
Secondary infection (n=600)			0.434	
Yes	160 (47.1)	41.7-52.5		1.13
No	114 (43.8)	37.7-50.1		1
Acute kidney failure (n=282)			0.14	
Yes	13 (54.2)	32.8-74.4		1.86
No	100 (38.8)	32.8-45.0		1
Hepatic cytolysis (n=424)			0.031	
Yes	45 (55.6)	44.1-66.6		1.70
No	145 (42.3)	37.0-47.7		1

Independent predictors of Covid-19 mortality: binary logistic regression

Table 5 reports the final binary logistic regression model used to adjust on confounding factors to predict mortality among in-patients with Covid-19. The independent predictors of Covid-19 mortality are age greater than 60 years old, p<0.001; the use of invasive ventilation during hospitalization, p<0.001; the stay in the resuscitation unit, p<0.001; and acute respiratory distress syndrome (ARDS), p<0.001.

**Table 5:** Logistic regression for the relationship between clinical characteristics and death among in-patients with Covid-19 aOR: adjusted odds ratio; 95% CI: 95% confidence interval

Variable	P-value	aOR	95% CI
Age of >60 years	0.000	4.90	2.31-10.38
Invasive ventilation	0.000	9.60	3.14-29.31
Stay in the resuscitation unit	0.000	5.09	2.43-10.67
Acute respiratory distress syndrome	0.000	6.49	3.34-12.61
Constant	0.000	0.008	

## Discussion

In the present study, we included 615 Covid-19 in-patients and followed them throughout the hospitalization period and then 30 days after discharge. We found that age of >60 years old (p<0.001 and adjusted odds ratio {aOR}=4.90), the use of invasive ventilation (p<0.001 and aOR=9.60), the stay in the resuscitation unit (p<0.001 and aOR=5.09), and acute respiratory distress syndrome (p<0.001 and aOR=6.49) were independent predictors of mortality from Covid-19.

Patients in our cohort were more likely to be male (62.1%). This is consistent with the results of clinical observations reported elsewhere, since in the majority of studies conducted in different countries, patients hospitalized for Covid-19 were predominantly male [8,9].

Univariate analysis in our study showed that male gender was significantly associated with death (p<0.008 and brut relative risk {_b_RR}=1.57). Male gender has been identified as a risk factor for Covid-19 mortality since the onset of the pandemic [4,8,10], and its predominance has been noted in studies [10]. These sex differences in Covid-19 outcomes already exist in the context of viral infections. It is due to the difference between the two genders in the hormonal and genetic regulation of immune responses [11].

The mean age in this study was 62.53±13.67 years old, and the median age was 64 years old. This result does not diverge from the results shown in other studies. Merugu et al. found a mean age of 63.13±17.8 years old [12]. Khamis et al. found a mean age of 54±16 years old [9]. In Turkey, the team of Aksel et al. found a median patient age of 59.5 years old [13]. Our univariate analysis results showed that advanced age was significantly associated with death (p<0.001 and _b_RR=4.90). We note that the frequency of death increases with age. We underline that in the multivariate analysis, we used the age variable in two categories (<60 years and ≥60 years) because below 60 years old, the relationship between age and death was not statistically significant. We note that in several studies, advanced age has been also identified as a risk factor [3,4,10] and predictor [9,14] of death from Covid-19.

We have noted that active smokers in our cohort were infrequent (5.7%), which is consistent with the literature, as the percentage of active smokers among patients hospitalized for Covid-19 has been found low in studies [15,16]. In a systematic review studying the prevalence of active tobacco smoking among patients hospitalized for Covid-19 in China, pooled analysis revealed a low prevalence (6.5%) compared with the prevalence of smoking in China (26.6%). This finding prompted the investigators to hypothesize that nicotine might have some beneficial effect on the disease [16]. Regarding the relationship between smoking and Covid-19 mortality, study results appear to be conflicting. In our study, we did not find such an association (p=0.635). In a large prospective cohort study including 4,244 critical patients, investigating predictors of 90-day mortality, the results showed no association between active smoking and Covid-19 mortality [15]. Aksel et al. also found no relationship between smoking (current or quit) and Covid-19 mortality [13]. On the other side, in a meta-analysis exploring predictors of mortality, smoking was associated with a high risk of in-hospital death (pooled odds ratio {p-OR}=1.6) [17]. In addition, Alharthy et al. studied 28-day mortality in critical patients with Covid-19, and multivariate regression analysis showed that active smoking (odds ratio {OR}: 3 and 95% CI: 2.51-3.66) was an independent predictor of mortality [14]. In contrast, in a large case-control study of comorbidities associated with high risk of Covid-19 disease, smoking was found to have a protective effect [18].

Our results showed that only 80 out of 615 patients were vaccinated. This proportion may seem low, but it is due to the fact that the vaccination campaign against SARS-CoV-2 was launched in Morocco on January 28, 2021. As a result, 360 patients were included in the study before the vaccination campaign even began. As of January 28, 2021, we included 255 in-patients, and 32.4% of them were vaccinated. In a systematic review including 13 randomized trials on the safety and efficacy of 11 Covid-19 vaccines, it was found that in 10 trials, the seroconversion rate of individuals at 28 days was greater than 80%. Four of six trials comparing single- and double-dose vaccination demonstrated that double-dose vaccination provided a higher immune response than single-dose vaccination [19]. Our results didn't show a significant association between vaccination status and Covid-19 mortality even after adjustment on confounding factors, even if all vaccinated patients followed the vaccination schedule recommended by the Ministry of Health.

In our study, the median time from symptom onset to hospitalization was eight days, indicating that patients' condition worsened during the transition from the first to the second week of illness. Huang et al. found that the median duration from symptom onset to first hospital admission was seven days, and the median time from illness onset to dyspnea was eight days [6]. In parallel, a Moroccan study conducted by the team of El Aidaoui et al. found a median time of seven days from onset of illness to hospital admission [20].

Regarding comorbidities, we observed in our cohort a predominance of diabetes (50.7%), anemia (39.6%), and hypertension (35.7%). In a large case series including 5,700 patients from the New York City area, the most common comorbidities were hypertension (56.6%), obesity (41.7%), and diabetes (33.8%) [21]. Comorbidities such as cardiovascular disease [4,9], hypertension [10], diabetes [10], pulmonary disease [8], and liver disease [9] were found to be risk factors for Covid-19 mortality. In our results, we did not find a significant association between comorbidities and risk of death. We explain that by that fact, the Ministry of Health of Morocco recommended hospitalization for Covid-19 patients with at least one risk factor including comorbidities even for asymptomatic and mild forms of the disease. This conservative approach was adopted at a national level in order to follow these patients closely and manage them early in case of aggravation.

Concerning radiological examinations, the CT scan performed at admission showed that 91.5% of the patients had lung involvement greater than 25%. We noted that the risk of death increased with the degree of lung involvement (p<0.001). In this regard, studies have shown that chest CT scan and lesion scoring can predict mortality [22]. Francone et al. [23] were interested in the existence of a correlation between the CT score and the short-term prognosis. They calculated the semiquantitative CT score, proposed by Pan et al. [24]. The results of the study showed that a CT score of ≥18 was associated with a high risk of mortality, in both univariate (HR: 8.33; 95% CI: 3.19-21.73; p<0.0001) and multivariate analysis (HR: 3.74; 95% CI: 1.10-12.77; p<0.0348) [23]. In another study conducted in Iran on 121 patients, the researchers calculated a score called the CT severity score. The results of the study showed that a CT severity score of >8 was associated with a high risk of mortality (OR: 5.29; 95% CI: 1.44-19.32; p<0.012) [25]. This is consistent with our results, which show that the greater the global lung damage, the higher the risk of death.

On admission, the most common symptoms observed in patients were dyspnea (82.2%), cough (80.3%), and fever (76.8%). This result is consistent with the literature, as observational studies of in-patients since the beginning of the epidemic in China have shown that the two most common symptoms were fever and cough [6,26], followed by dyspnea [26] and/or fatigue [6].

Biological tests revealed lymphopenia (lymphocytes of <1500), high blood creatinine, D-dimer of >500 µg/l, C-reactive protein (CRP) of >130 mg/l, ferritin of >300 µg/l, and lactate dehydrogenase (LDH) of >500 U/l in 78.5%, 33.5%, 82.7%, 47.7%, 77.3%, and 49.7% of the cases, respectively. Lymphopenia has been reported in several studies [4,27]. Li et al. [4] and Guan et al. [27] found lymphopenia in 90.2% and 83.2% of patients, respectively. Other biological parameters (CRP, ferritin, D-dimer, LDH, and creatinine) were found to be elevated in patients with Covid-19 in several research studies [4,12]. We found laboratory parameters at admission related to the risk of death, such as lymphocytes of <1500 (p<0.001), D-dimers of >500 µg/l (p<0.007), CRP of >130 mg/l (p<0.001), high blood creatinine (p<0.013), and LDH of >500 U/l (p<0.001). This is in accordance with the literature, as Zhou et al. found that a D-dimer level greater than 1 μg/ml on admission was associated with a high risk of in-hospital death [3]; also, Alharthy et al. found that high D-dimer and lactate levels were predictive of mortality [14]. The survival analysis by Li et al. also showed that elevated lactate dehydrogenase levels were associated with death in patients with severe Covid-19 [4]. An international multicenter study identified blood creatinine (≥1.2 mg/dL) and elevated CRP levels as primary risk factors for mortality [28]. CRP has been identified in other studies as an independent predictor of mortality [13]. We did not find a significant relationship between elevated serum ferritin and death, although this relationship has been demonstrated in several studies [9].

The treatment protocol adopted for all patients was that set by the Ministry of Health. This protocol contains the combination of azithromycin+hydroxychloroquine, vitamin C, vitamin D, and zinc. And during hospitalization, other drugs are added such as low-molecular-weight heparin (LMWH), corticoids, and additional antibiotics, depending on the indication. Patients who did not receive the combination "hydroxychloroquine+azithromycin" had a higher risk of death (p<0.001). Patients who received corticosteroids also had a higher risk of death (p<0.001 and _b_RR=5.08). Li et al. found in their study that high-dose corticosteroid use was related to death in patients with severe Covid-19 [4]. We did not find an association between LMWH therapy and death, but patients who were on anticoagulants before admission had a lower risk of death (p<0.005 and _b_RR=0.46). Those patients were on anticoagulants as they were already presenting a chronic disease requiring medication. This is consistent with the fact that Covid-19 was also a vascular disease with high thrombotic risk [29]. The observational study by Nadkarni et al. of 4,389 patients with Covid-19 showed the benefit of anticoagulant therapy and its association with low in-hospital mortality, whether at prophylactic or curative doses [30].

The most common complications observed in our cohort were secondary infection (56.9%), acute respiratory distress syndrome (26.1%), and pulmonary embolism (19.8%). In the study by Li et al., the three most observed complications in patients were acute respiratory distress syndrome (38.3%), hyperglycemia (33.2%), and cardiac injury (21.7%) [4]. In their work, Khamis et al. studied in-patient mortality and found that mortality was associated with acute respiratory distress syndrome and ICU admission [9].

Strengths and limitations

The strengths of our study are the large number of patients in the cohort, the mixed design of the study that allows for representativity over time through inclusion over a long duration, the important prospective part that reduced missing data, and the follow-up of patients 30 days after discharge that prevented the death misclassification bias. In addition, we used the ISARIC-WHO Case Report Form (modified), which allows comparability of data with other studies. Moreover, our study is the only one in Morocco to have investigated mortality in Covid-19. The limitations of our work lie in the retrospective part of the study design (missing data) and in the fact that it is a single-center study.

## Conclusions

In this cohort study that focused on Covid-19 in-patient's mortality, we found that age of >60 years, the use of invasive ventilation, the stay in the resuscitation unit, and acute respiratory distress syndrome were independent predictors of Covid-19 mortality. These outcomes can be used to improve the clinical management of patients.
